# Bruton’s tyrosine kinase (BTK) mediates resistance to EGFR inhibition in non-small-cell lung carcinoma

**DOI:** 10.1038/s41389-021-00345-8

**Published:** 2021-07-27

**Authors:** Chi-Tai Yeh, Tzu-Tao Chen, Pamungkas Bagus Satriyo, Chun-Hua Wang, Alexander T. H. Wu, Tsu-Yi Chao, Kang-Yun Lee, Michael Hsiao, Liang-Shun Wang, Kuang-Tai Kuo

**Affiliations:** 1 Department of Medical Research and Education, Shuang Ho Hospital, Taipei Medical University, New Taipei City, Taiwan; 2 Division of Hematology & Oncology, Department of Medicine, Shuang Ho Hospital, Taipei Medical University, New Taipei City, Taiwan; 3 Department of Medical Laboratory Science and Biotechnology, Yuanpei University of Medical Technology, Hsinchu City, Taiwan; 4 Division of Pulmonary Medicine, Department of Internal Medicine, Shuang Ho Hospital, Taipei Medical University, New Taipei City, Taiwan; 5 Division of Pulmonary Medicine, Department of Internal Medicine, School of Medicine, College of Medicine, Taipei Medical University, Taipei, Taiwan; 6 Faculty of Medicine Public Health and Nursing, Universitas Gadjah Mada, Yogyakarta, Indonesia; 7 Faculty of Medicine Public Health and Nursing, Department of Pharmacology and Therapy, Universitas Gadjah Mada, Yogyakarta, Indonesia; 8 Department of Dermatology, Taipei Tzu Chi Hospital, Buddhist Tzu Chi Medical Foundation, New Taipei City, Taiwan; 9 School of Medicine, Buddhist Tzu Chi University, Hualien, Taiwan; 10 The Ph.D. Program for Translational Medicine, College of Medical Science and Technology, Taipei Medical University, Taipei, Taiwan; 11 Graduate Institute of Clinical Medicine, College of Medicine, Taipei Medical University, Taipei, Taiwan; 12 Genomics Research Center, Academia Sinica, Taipei, Taiwan; 13 Division of Thoracic Surgery, Department of Surgery, Shuang Ho Hospital, Taipei Medical University, New Taipei City, Taiwan; 14 Division of Thoracic Surgery, Department of Surgery, School of Medicine, College of Medicine, Taipei Medical University, Taipei, Taiwan

**Keywords:** Non-small-cell lung cancer, Prognostic markers

## Abstract

Epidermal growth factor receptor (EGFR) tyrosine kinase inhibitors (TKIs) are current standard of care for patients with EGFR mutation and metastatic non-small-cell lung carcinoma (NSCLC), but most patients using EGFR TKIs acquire resistance later. So, overcoming resistance of EGFR TKIs has become an important issue in the treatment of NSCLC. Previously, therapeutics targeting Bruton’s tyrosine kinase (BTK) have been successful in treating several hematologic malignancies. However, the role of BTK in NSCLC is still unknown. In this study, by examining surgical specimens from 80 NSCLC patients and their clinicopathologic parameters, we found significant correlation between high BTK expression and tumor differentiation, p-stage, lymph node metastatic status, maximum tumor size, and poor prognosis of patients. Using two NSCLC cell lines A540 and PC9, we demonstrated that BTK^pos^ cells exhibited more stemness (OCT4, SOX2) and EMT (E-Cadherin, Slug) markers than BTK^neg^ cells. Knockdown of BTK sensitized the NSCLC cells to Gefitinib. Meanwhile, the second-generation BTK inhibitor Acalabrutinib effectively suppressed SOX2, STAT3/JAK2/Akt axis and potentiated the anti-proliferative effect of Gefitinib and Osimertinib in NSCLC cells, including the T790M H1975 cells. Furthermore, Acalabrutinib and Osimertinib combination exhibited significant tumor growth inhibition of H1975-derived tumors in vivo. Our findings suggested that BTK mediates stemness and EMT properties, and inhibition of BTK potentiates the effect of Gefitinib and Osimertinib in NSCLC cells resistant to TKI. This implies a new approach to treat the NSCLC patients with resistance to previous TKI treatment.

## Introducton

Lung cancer is the leading cause of cancer death worldwide, with an estimated global mortality of nearly 1.59 million in 2012 [1]. Non-small-cell lung carcinoma (NSCLC) accounts for 83% of all lung cancers, with >60% of patients diagnosed at stage III or IV [2]. With the advances in various treatments including target therapies, the survival of patients with NSCLC of all stages has improved remarkably from <20% to ~23% [2, 3]_._ Among the target therapies, tyrosine kinase inhibitors (TKIs) working against epidermal growth factor receptor (EGFR) with different generations were most widely used [4–6]. However, patients treated with EGFR TKIs eventually acquired resistance largely as a result of the development of the EGFR T790M mutation and subsequent disease progression [7]. Therefore, alternative targets are urgently required to treat these EGFR-TKI-resistant patients.

A nonreceptor tyrosine kinase, Bruton’s tyrosine kinase (BTK), has received considerable attention for its efficacy as a drug target for B-cell malignancies [8, 9]. BTK is a member of the TEC kinase family, a group of nonreceptor kinases comprising five members, namely TEC, BTK, bone marrow-expressed kinase or epithelial and endothelial tyrosine kinase (ETK), TXK or redundant resting lymphocyte kinase, and interleuki-2 inducible T-cell kinase (ITK) [10]. BTK plays a critical role in the B-cell receptor signaling pathway [11]. A key feature of BTK is its interaction with the PI3K/Akt signaling pathway and function as the upstream of NF-kB and ERK, thereby affecting the proliferation, survival, and differentiation of lymphoma cells [12, 13]. These characteristics mean that BTK is ideal for drug development for the purpose of disrupting two major oncogenic pathways simultaneously.

BTK is mainly expressed in bone marrow–derived cells, and most studies have focused on BTK in terms of lymphoma, though others have suggested that BTK plays a vital role in the oncogenic process of solid tumors, such as colon cancer, ovarian cancer, and glioma [14–16]. Furthermore, BTK is a therapeutic target of stem-like cells from multiple myeloma because its elevated expression leads to the upregulation of key stemness genes, including OCT4, SOX2, NANOG, and MYC [17]. As for lung cancer, the BTK inhibitor ibrutinib effectively suppresses the proliferation of certain EGFR mutant lung cancer cells through the inhibition of the autophosphorylation of EGFR [18]. Studies have also revealed that ETK is responsible for mediating drug resistance in small-cell lung cancer [19]. Because BTK shares similarities with ETK, BTK may also be a potential target for lung cancer. However, few studies have investigated the biological role of BTK in tumorigenesis or explored the effects of BTK inhibitors in NSCLC.

In this study, we evaluated the expression of BTK in NSCLC tumor tissues and cell lines, analyzed its clinicopathologic significance in patients with NSCLC, and investigated its functional roles in NSCLC tumorigenesis, with focus on epithelial–mesenchymal transition (EMT) and stemness. In addition, we treated the EGFR-TKI-resistant H1975 cells with Acalabrutinib, a more specific second-generation BTK inhibitor, and evaluated its effectiveness.

## Methods

### Targeted drugs, cell Lines, and culture

Three target drugs including EGFR inhibitor Gefitinib (ZD1839), second-generation BTK inhibitor Acalabrutinib (ACP-196), and third-generation epidermal growth factor receptor tyrosine kinase inhibitor Osimertinib (AZD9291) were purchased from Selleckchem, Inc (Houston, TX, USA). Stock solutions of 10 mM in sterile dimethyl sulfoxidewere (DMSO). The stocks of each drug were stored at −20 °C until use. The human lung adenocarcinoma cell lines A549 and H1975 were purchased from American Type Culture Collection (ATCC, Manassas, VI, USA). The human lung adenocarcinoma cell lines PC9 and PC9/GR were kindly provided by Dr. Michael Hsiao, Genomics Research Center, Academia Sinica, Taipei, Taiwan. Characteristically, A549 cells have wild type *EGFR*, PC-9 cells harbor an *EGFR* exon 19-deletion and are highly sensitive to *EGFR* TKI. H1975 cells carry the *EGFR* L858R-T790M mutation and are resistant to *EGFR* TKI. PC-9/GR acquired Gefitinib resistance by chronic exposure of PC-9 cells to medium with increasing Gefitinib concentrations. Briefly, PC9/GR NSCLC cells were exposed to 10 nM Gefitinib in medium containing 10% FBS, and the concentration was increased in a stepwise manner. Cells that were able to grow in 1 µM Gefitinib were obtained 6 months after the initial exposure. A549, PC-9 and H1975 cell lines were maintained in in RPMI-1640 media (Gibco, Carlsbad, CA, USA) supplemented with 10% fetal bovine serum (FBS; Gibco-Invitrogen) and 1% penicillin/streptomycin (Gibco) at 37 °C in a humidified atmosphere of 5% CO_2_.

### Immunohistochemical staining and scoring

This study was conducted in a cohort of 80 NSCLC patients in Taipei Medical University - Shuang Ho Hospital (TMU-SHH). It was approved by the institutional human research ethics review board (TMU-JIRB N201801066) of Taipei Medical University. Clinical samples from NSCLC patients were fixed in 10% formalin, embedded in paraffin, deparaffinized and then rehydrated. For immunohistochemical (IHC) staining, rehydrated sections were subjected to antigen retrieval and their endogenous peroxidase activity blocked for 30 min in 1% H_2_O_2_ /PBS solution. After blocking, the slides were exposed to BTK (D3H5 rabbit monoclonal antibody; Cell Signaling Technology, 1:200) at 4 °C overnight, washed and incubated in biotinylated link universal antiserum for 1 h at room temperature. Slides were then rinsed, and stain was developed using the chromogen, 3, 3- diaminobenzidine hydrochloride. Finally, sections were rinsed with ddH_2_O and counterstained with hematoxylin. The stained tissues were scored separately by two independent pathologists (Dr. Wei-Hwa Lee and Dr. Shiou-Fu Lin), blinded to each other’s results and all clinical data. Where there was a discrepancy, a consensus diagnosis was subsequently reached by both pathologists using a multiheaded microscope. The percentage of stained area to the selected field was recorded in a 5% interval, ranging from 0 to 100%. The staining intensity was graded into 3 categories (absent or weak, 1; moderate, 2; strong, 3). Quick score (Q-score) was derived from the product of percentage (P) of tumor cells with characteristic IHC staining (1: score <25%, 2: 25–49%, 3: 50–74%, 4: score ≥75%) and the intensity (I) of IHC staining (1–3) (Q = P × I; maximum = 12). We further analyzed the NSCLCs IHC scores of 80 patients and reciprocal receiver operating characteristic (ROC) curves regarding overall survival were generated. ROC curve analysis showed the optimal cut-off point is 8.0 with 0.694 sensitivity and 0.742 specificity, as shown in Supplementary Fig. S1. So Q-score = 8 was used as a cut-off value to divide the patients into two groups, with Q-score ≥ 8 as BTK high and Q-score < 8 as BTK low.

### Immunoblotting analysis

Western blotting was performed using standard methods. Cells were washed with PBS and then lysed in radioimmunoprecipitation assay lysis buffer; cellular protein lysates were isolated using a protein extraction kit (Qiagen, Carlsbad, CA, USA) and quantified using the Bradford protein assay kit (Carlsbad, CA, USA). After preparing the whole cell lysate, the proteins were separated using sodium dodecyl sulfate–polyacrylamide gel electrophoresis, transferred to a polyvinylidene fluoride (PVDF) membrane, and blocked with Tris-buffered saline and skimmed milk. These PVDF membranes were then probed with respective primary antibodies, followed by the secondary antibody. The commercial antibodies are described in Supplementary Table S1. Images were captured and analyzed using the UVP BioDoc-It system (Upland, CA, USA).

### Real-time polymerase chain reaction

Total RNA was isolated from lung cancer cell lines using a Trizol reagent (Invitrogen) according to the manufacturer’s instructions. Reverse transcription reactions were performed with the Transcriptor First Strand cDNA Synthesis Kit (Roche, Indianapolis, IN, USA). Real-time polymerase chain reaction (RT-PCR) was performed using a Rotor-Gene Q (Qiagen). The reaction was subjected to 42-cycle amplification at 95 °C for 20 s, at 60 °C for 20 s, and at 72 °C for 25 s. Relative mRNA expression of the selected genes was normalized to HPRT and quantified using the ddCT method. The sequences of the RT-PCR primers are listed in Supplementary Table S2.

### Cell stable transfection

The information of a BTK plasmid (RG211582 OriGene, Rockville, MD, USA) was used to design polymerase chain reaction primers. Cell transfection was performed using Lipofectamine 2000 (Invitrogen), following the manufacturer’s protocols. An 8 μg empty plasmid (pCMV-MCS-N1, empty vector control plasmid DNA, GenBank accession number U55762) or a BTK plasmid was used. The DNA-lipofectamine reagent complexes sat at room temperature for 30 min. The mixture was then added to the well and mixed gently by rocking the plate back and forth, and reagent complexes were not removed following transfection. The cells were incubated at 37 °C in a CO_2_ incubator for 24 h and then assayed for transgene expression.

### BTK knockdown procedure

The loss of function of BTK in the lung cancer cell lines was studied using commercially available systems. BTK gene-silencing shRNA sets (Expression Arrest GIPZ Lentiviral shRNA) were purchased from Thermo Fisher Scientific (Bartlesville, OK, USA). A6 (clone ID, V2LHS-89195) and B10 (V3LHS-639151) clones were used to silence BTK expression, with a nonsilencing verified negative control (RHS4346) acting as the control. The production of lentiviral particles for loss-of-function studies was conducted according to the manufacturer’s instructions.

### Cell migration and invasion assay

NSCLC cells were cultured to 90% confluence in six-well plates. For assessment of cell migration, images were captured under a microscope at 0 and 24 h. For invasion assay, Matrigel-coated transwell inserts with micropore membranes (BD Biosciences, San Jose, CA, USA) were placed in 24-well plates. In the upper chamber, 3 × 10^4^ cells were plated in 100 μL of medium containing 1% FBS, and the lower chamber was filled with 600 μL of complete growth medium. Cells were incubated in a 5% CO_2_ humidified atmosphere at 37 °C for 48 h. The noninvading cells were scraped from the upper chamber of each insert with a cotton swab, and the invaded cells attached to the lower surface of the insert membrane were incubated in 0.1% crystal violet at 37 °C for 30 min; they were then washed twice with PBS and viewed under a microscope.

### Flow cytometry

After dissociation into a single-cell suspension from the NSCLC cell culture, the suspension was transferred into a 15-mL falcon tube. The cells were washed with 10 mL PBS (containing 10% FBS), followed by centrifugation at 200 × *g* for 10 min. The supernatant was removed and counted using a hemacytometer. BTK staining was conducted using the BTK monoclonal antibody according to the manufacturer’s instructions. For separation of the NSCLC cells with BTK high and low expression during fluorescence-activated cell sorting (FACS; BD Biosciences, Franklin Lakes, NJ, USA), we used a diluted concentration of antibody at 1:1000. BTK high and low expression cells were isolated through FACS. After sorting, they were washed with sterile PBS three times to remove all serum in the cells, with each washing centrifuged at 200 × *g* for 10 min. After the NSCLC cell classification, BTK-enriched cells were tested for their stemness and differentiation potential in vitro. BTK lung cancer cell populations were sorted through FACS, and the cells measured using a FACS-Canto-II (BD Biosciences, Franklin Lakes, NJ, USA). The data were analyzed using FlowJo software (Tree Star, Ashland, OR, USA).

### Immunofluorescence staining

PC-9 cells with BTK overexpression (BTK^pos^) and the related control were plated in six-well chamber slides for 24 h for immunofluorescence analysis. The cell sample was fixed with 2% paraformaldehyde and probed with primary antibodies against SOX2 (1:500; 79351, mouse monoclonal antibodies; Abcam, Cambridge, UK), pSTAT3 (1:500; Phospho-Stat3 [Tyr705] 76315, rabbit monoclonal antibodies; Cell Signaling, Danvers, MA, USA). A fluorophore-conjugated secondary antibody was added to verify the positive signal using a Zeiss Axiophot (Carl Zeiss, Jena, Germany) fluorescent microscope. The nuclei of viable cells were detected through 4′,6-diamidino-2-phenylindole staining.

### Lung cancer spheroids self-renewal assay

The cells were transferred to serum-free low-adhesion culture plates containing DMEM and F-12 with N2 supplement (Invitrogen), 20 ng/mL epidermal growth factor, and 20 ng/mL mimic-fibroblast growth factor (stem cell medium; PeproTech, Rocky Hill, NJ, USA) for 2 weeks to allow for tumor sphere formation. The spheres were then counted under microscope, and their formation efficiency calculated as the ratio of the sphere number to the planted cell number.

### Colony formation assay

We suspended 500 cells/cm [2] in 0.3% agarose with MammoCult medium (Stem Cell Technologies, San Jose, CA, USA) and a 0.8% agar base layer. The culture was covered with 0.5 mL of MammoCult medium and cultured for 14 days. For quantification, the wells were imaged using a microscope, and the colonies were analyzed using ImageJ software.

### Cell viability test and calculation of the combination index

The effects of Acalabrutinib and Gefitinib on cell proliferation were assessed using a sulforhodamine B (SRB; Sigma Aldrich, St. Louis, MO, USA) assay. PC-9 or H1975 cells were seeded in 96-well plates (8 × 10^5^ cells/well) and treated with Acalabrutinib or Gefitinib alone or in combination at different concentrations for 48 h, respectively. After respective drug treatments, the relative cell number was estimated using the SRB reagent according to the manufacturer’s protocol. The stock of Acalabrutinib and Gefitinib was prepared by dissolving 20 mg/mL of the mixture in dimethyl sulfoxide. The stocks of each drug were stored at −20 °C until use. Using CompuSyn software, the half-maximal inhibitory concentration (IC_50_) values of different cell lines were calculated as described in the “PC Software and User’s Guide” on the ComboSyn website (http://www.combosyn.com). The combination index (CI) was calculated with CompuSyn software, with a CI value <1 representing synergism.

### Tumor xenograft studies

Female NOD/SCID (6 weeks old) were purchased from BioLASCO (BioLASCO Taiwan Co. Ltd., Taipei, Taiwan) and maintained under specific pathogen-free condition with free access to standard chow and water. Acalabrutinib (ACP-196) and Osimertinib (AZD9291), purchased from Selleckchem, Inc (Houston, TX, USA), were suspended in 0.5% w/v methylcellulose/0.1% v/v Tween 80 for in vivo study. The mice were subcutaneously inoculated with 1 × 10^6^ H1975 cells suspended in 100 μL of serum-free medium. Each group was randomly divided into four subgroups (*N* = 5 per group): vehicle control, Osimertinib only, Acalabrutinib only and combination (Osimertinib + Acalabrutinib). The treatment regimens were defined as: 20 mg/kg Osimertinib (i.p. injection, 5 times/week); 25 mg/kg Acalabrutinib (i.p injection, 5 times/week); the combination group (20 mg/kg Osimertinib + 25 mg/kg Acalabrutinib, 3 times/week) while the vehicle group received PBS injection. Tumor volume (mm [3]) was measured weekly using an established formula where the tumor volume = (*L* × *W* [2])/2, where *L* and *W* represent the longest and shortest diameters, respectively. The mice were humanely killed at the end of the experimental period, and tumors were collected for further analyses. All animal experiments were approved by the Institutional Animal Care and Use Committee of Shuang Ho Hospital (LAC-2018–0547).

### Statistical analysis

All assays were performed in triplicate. Data are expressed as mean ± standard error of the mean. All statistical analyses were performed using GraphPad prism (v.6.0. GraphPad Software, San Diego, CA, USA). Survival analysis for both patients and xenograft animals was performed using the Kaplan–Meier (KM) plots and logrank test. The correlation between BTK expression and the clinicopathological parameters was assessed using the *χ*^2^ test and bivariate analysis. For comparisons between two groups, including patients and xenograft animals, a Student *t* test was employed and, for more than two groups, one-way analysis of variance (ANOVA) was used. For a *p* value of <0.05 was considered statistically significant. *p < 0.05, **p < 0.01.

## Results

### BTK expression correlates with clinicopathologic parameters and high expression of BTK is significantly associated with poor prognosis of NSCLC patients

**Fig. 1:**
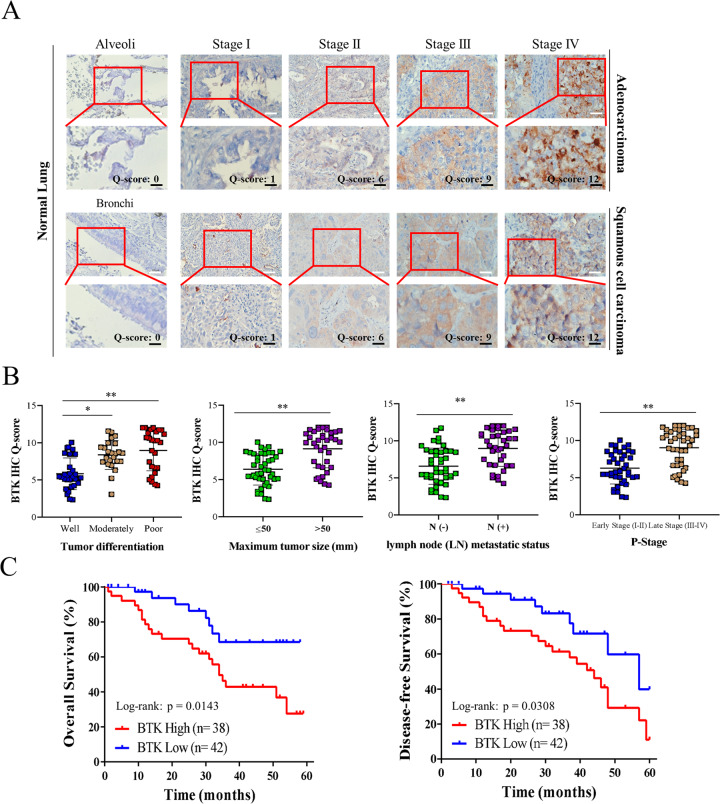
BTK overexpression correlates with advanced stage status and worse overall survival (OS), disease-free survival (DFS) in NSCLC patients. **A** Representative photos of immunohistochemical staining for BTK in lung adenocarcinoma and squamous cell carcinoma with cases of different stages. The alveoli tissues were used as the control for adenocarcinoma, while bronchi tissues were used as the control for squamous cell carcinoma. The enlarged view of the selected area was shown below. scale bar 25 μm **B** Increased BTK expression correlated significantly with tumor differentiation, maximum tumor size, lymph node (LN) metastatic status, and pathologic (P-)stage in NSCLC. **C** The 5-year OS and DFS rates were 67.5, 38.7, and 26.8%, 10.3% in BTK-low (*n* = 42) and BTK-high (*n* = 38) NSCLC patients, respectively (both *p* < 0.05). Q-score ≥ 8 indicated BTK-high and Q-score < 8 indicated BTK-low. *p < 0.05, **p < 0.01.

Representative images of BTK IHC at each staining intensity level, including adenocarcinoma and squamous cell carcinoma, are presented in Fig. 1A. The alveoli tissues were used as the control for adenocarcinoma, while bronchi tissues were used as the control for squamous cell carcinoma. Statistically significant associations were found between BTK IHC score and tumor differentiation, maximum tumor size, lymph node metastatic status and pathologic stage (Fig. 1B). Using Q-score = 8 as the cut-off value, the 5-year overall survival (OS) rates in the BTK low (Q-score < 8) and BTK high (Q-score ≥ 8) groups were 67.5% and 26.8%, respectively, whereas the 5-year disease-free survival (DFS) rates in the BTK low and BTK high groups were 38.7% and 10.3%, respectively (both *p* < 0.05; Fig. 1C). Higher BTK expression was significantly associated with poor prognosis of NSCLC patients. Meanwhile, when we used the same standard to compare the clinicopathologic parameters, as shown in Table 1, we found that tumor differentiation, tumor size, lymph node metastatic status, and pathologic stage remained significantly different between BTK high and BTK low group. Moreover, cell type was also a significant factor, with BTK high less frequent in squamous cell carcinoma. We then used Cox proportional hazard analysis to identify the risk factors including age, gender, TNM stage, subtype plus BTK expression for overall survival, and found that BTK expression and age were independent factors for overall survival in NSCLC patients (Supplementary Table S3).

**Table 1:** Patient clinicopathological characteristics of the NSCLC cohort.

Clinicopathological variables	No. (*n* = 80)	High expression	Low expression	*x*^2^	*p*-value
		BTK			
Age, years					
≤65	37	15 (39.5)	22 (52.4)	1.337	0.248
>65	43	23 (60.5)	20 (47.6)	–	–
Gender					
Male	49	22 (57.9)	27 (64.3)	0.343	0.558
Female	31	16 (42.1)	15 (35.7)	–	–
Differentiation					
Well/moderately	53	21 (55.3)	32 (76.2)	3.908	0.048
Poor	27	17 (44.7)	10 (23.8)	–	–
Tumor size (mm)					
≤50	43	13 (34.2)	30 (71.4)	11.116	<0.001
>50	37	25 (65.8)	12 (28.6)	–	–
Lymph node metastasis					
N0	44	15 (39.5)	29 (69.0)	7.050	0.008
N1–N2	36	23 (60.5)	13 (31.0)	–	–
Pathologic stage					
I + II	40	11 (28.9)	29 (69.0)	12.832	<0.001
III + IV	40	27 (71.1)	13 (31.0)	–	–
Subtypes					
Adenocarcinoma	52	29 (76.3)	23 (54.8)	4.074	0.044
Squamous cell carcinoma	28	9 (23.7)	19 (45.2)	–	–

### BTK-positive NSCLC cells possess characteristics of cancer stemness and EMT

**Fig. 2:**
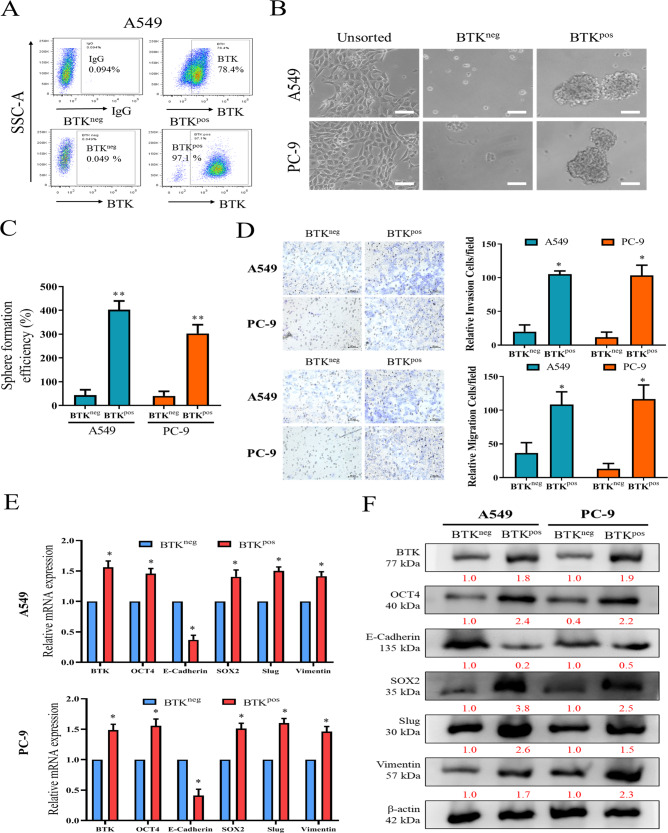
BTK-positive NSCLC cells possess the characteristics of stem cells and epithelial–mesenchymal transition (EMT). **A** Representative flow-cytometry plots of BTK for A549 cells showing the gating strategy for sorting. The efficiency was confirmed by flow cytometry following the sorting of BTK^pos^ and BTK^neg^ cells from A549 and PC-9 lung cancer cells. **B** Representative images of spheres (scale bar 50 μm) and **C** quantification of spheres in A549 and PC-9 lung cancer cells sorted for BTK. **D** Migration and invasion were increased in BTK^pos^ cells, as compared with BTK^neg^ cells, in both A549 and PC-9 cells. Scale bar 20 μm. **E** RNA expression of cancer stemness and EMT markers in BTK^pos^ cells and BTK^neg^ cells. **F** Protein expression of cancer stemness and EMT markers in BTK^pos^ cells and BTK^neg^ cells. *p < 0.05, **p < 0.01.

The IHC data suggested that BTK-high NSCLC tumors correlated with metastatic behaviors, thus we speculated that BTK^pos^ cells may be more “stem-like” and therefore evaluated the stemness characteristics of these BTK^pos^ cells. We first FACSorted the lung cancer cells for BTK (Fig. 2A) and determined their self-renewal capacity using sphere formation as a reveal. We observed that BTK^pos^ cells isolated from A549 and PC-9 lung cancer cells formed significantly more and larger spheres compared with BTK^neg^ cells (Fig. 2B, C), suggesting that BTK^pos^ cells are indeed enriched in cancer stem cells (CSCs). We then further isolated BTK^pos^ and BTK^neg^ cells for Transwell and Matrigel assays. The results showed that BTK^pos^ cells have significantly higher abilities of migration and invasion than BTK^neg^ cells in both A549 and PC-9 cells (Fig. 2D*)*. Meanwhile, gene expression (Fig. 2E) and western blot (Fig. 2F) confirmed that as compared with BTK^neg^ cells, BTK^pos^ cells expressed stronger stemness markers such as OCT4, SOX2, and mesenchymal markers including Slug, Vimentin, while the epithelial marker E-cadherin was downregulated.

### BTK drives EMT and tumor initiating cells properties by modulating the JAK2/STAT3/Akt axis in NSCLC Cells

**Fig. 3:**
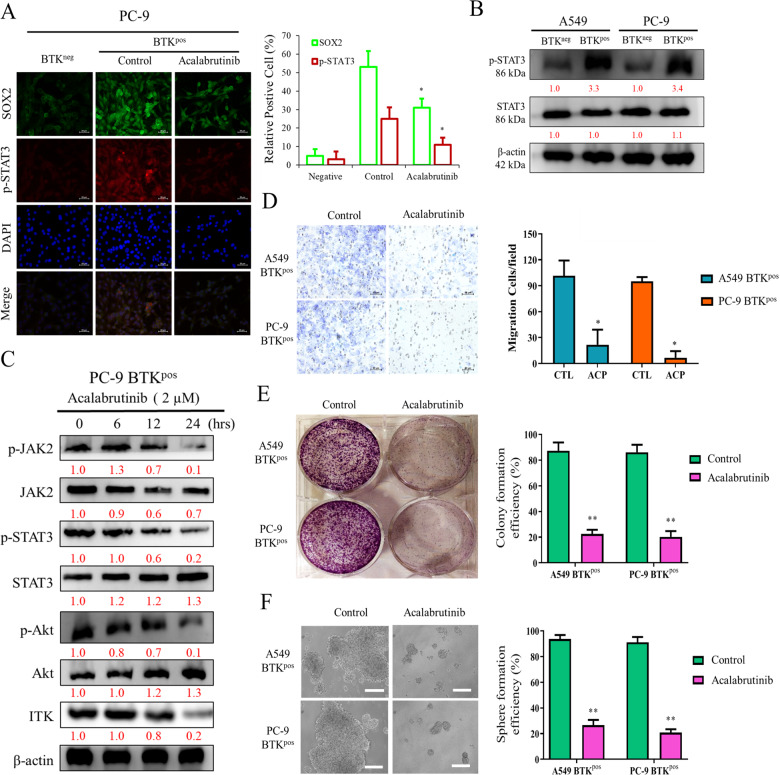
BTK regulates invasive phenotypes via the JAK2/STAT3 pathway in NSCLC cells. **A** Immunofluorescence study showed the level of SOX-2 and p-STAT3 were parallel in PC-9 cells with different status of BTK. Scale bar 50 μm. **B** p-STAT3 expression was upregulated in BTK^pos^ cells derived from both A549 and PC-9 cells. **C** Expression of p-STAT3, p-JAK2, p-Akt, and ITK in BTK^pos^ PC-9 cells were suppressed by Acalabrutinib in a time-dependent manner. Effect of Acalabrutinib on **D** cell migration (scale bar 20 μm), **E** colony formation, and **F** sphere formation (scale bar 50 μm) in BTK^pos^ A549 and PC-9 cells are present. CTL control. *p < 0.05, **p < 0.01.

Since it has been shown that the JAK2/STAT3 pathway was closely related to EMT [20, 21], plus our earlier data revealing the link between BTK and EMT, we further evaluated the relationship between BTK and the JAK2/STAT3 pathway. Our results showed that BTK overexpression (BTK^pos^) PC-9 cells have increased expression of p-STAT3 and SOX-2 in immunofluorescence study, whereas the BTK inhibitor Acalabrutinib abolished the upregulation of p-STAT3 and SOX-2 expression in these cells (Fig. 3A). Then we confirmed higher expression of p-STAT3 in BTK^pos^ cells from both A549 and PC-9 cell lines (Fig. 3B) and examined p-JAK2 and p-STAT3 expression in response to the treatment of Acalabrutinib in BTK^pos^ PC-9 cells. Our data showed that p-JAK2 and p-STAT3 levels were reduced upon Acalabrutinib treatment in a time-dependent manner. Additionally, p-Akt was also reduced in a time-dependent manner (Fig. 3C). We further investigated the effects of Acalabrutinib treatment in migration and stem cell characteristics of BTK^pos^ A549 and PC-9 cells. The results showed that Acalabrutinib treatment of BTK^pos^ A549 and PC-9 cells resulted in decreased degrees of migration (Fig. 3D). Moreover, colony formation (Fig. 3E) and sphere formation (Fig. 3F) of BTK^pos^ A549 and PC-9 cell were also significantly suppressed by the treatment of Acalabrutinib.

### BTK is involved in Gefitinib resistance and cancer stemness properties

**Fig. 4:**
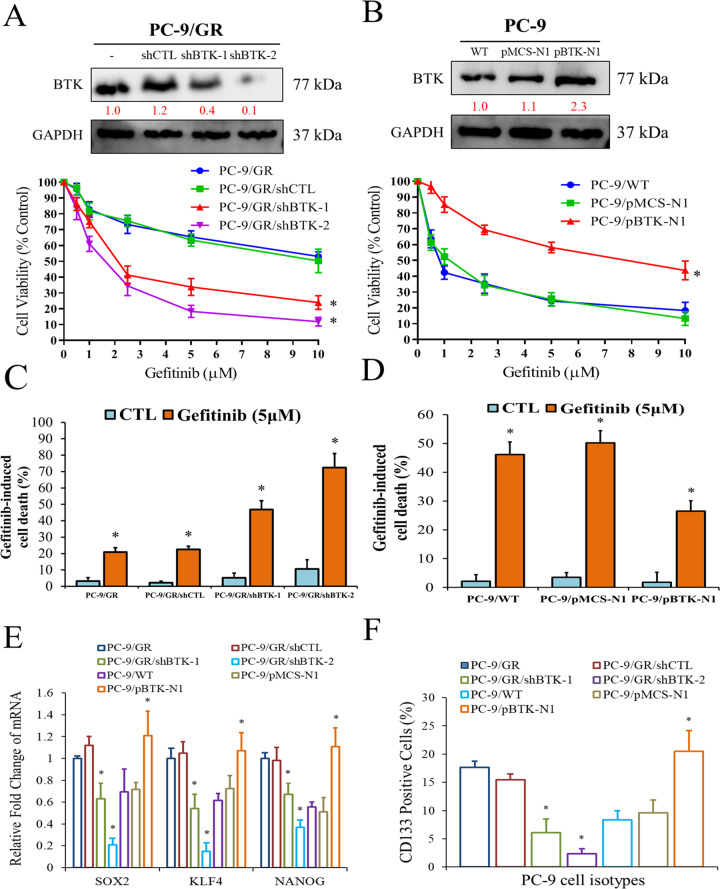
BTK is required for Gefitinib-induced cell death probably regulated by stemness. **A** Cell viability evaluated by SRB assay in PC-9/GR, PC-9/GR/shCTL, PC-9/GR/shBTK-1, and PC-9/GR/shBTK-2 cells. **B** Cell viability evaluated by SRB assay in PC-9 (wild type), PC-9/pMCS-N1 (vector control), and PC-9/pBTK-N1 (BTK overexpression) cells. **C** Cell apoptosis determined by flow cytometry after treatment of 5 μM Gefitinib in PC-9/GR, PC-9/GR/shCTL, PC-9/GR/shBTK-1, and PC-9/GR/shBTK-2 cells. **D** Cell apoptosis determined by flow cytometry after treatment of 5 μM Gefitinib in PC-9, PC-9/pMCS-N1, and PC-9/pBTK-N1 cells. **E** mRNA expression of KLF4, SOX2, and NANOG according to PC-9 cells with differed status of BTK expression and Gefitinib resistance. Total RNA was harvested for the analysis of mRNA by real-time RT-PCR. **F** CD133 + cells detected by flow cytometry according to PC-9 cells with different status of BTK expression and Gefitinib resistance. The results are shown as the means ± SD of three independent experiments, each performed in triplicate. CTL control, *p < 0.05.

To examine the role of BTK in Gefitinib resistance, we established two BTK-knockdown PC-9/GR cell lines with different degrees of BTK suppression (PC-9/GR was a cell line acquired EGFR resistance after continuous Gefitinib exposure) and showed that PC-9/GR/sh-BTK cells were significantly more sensitive to Gefitinib treatment and had more apoptotic cells upon Gefitinib treatment (Fig. 4A, C). Moreover, different degrees of BTK suppression resulted in significantly different degrees of drug sensitivity and cell apoptosis. On the other hand, overexpressing BTK in PC-9 cells (pBTK-N1 cells) resulted in more resistance to Gefitinib treatment (Fig. 4B) and correspondingly decreased Gefitinib-induced apoptosis as compared to the control pMCS-N1 cells (Fig. 4D). The downstream proteins associated with activated pathway in BTK overexpressing cells were shown in Supplementary Fig. S2. Overexpression of BTK increased the phosphorylation of STAT3 and activated the signaling pathway of NF-κB. Meanwhile, PC-9/GR/sh-BTK cells exhibited significantly lower levels of cancer stemness markers such as KLF4, SOX2, NANOG, and CD133, than its counterparts, whereas the pBTK-N1 cells expressed the highest levels of these markers (Fig. 4E, F). Based on the above results, the expression of BTK may play an important role in regulating the stemness and cell viability of lung cancer cells.

### BTK inhibitor Acalabrutinib induces apoptosis in A549 (EGFR wild type), H1975 (EGFR T790M), and PC-9 (EGFR with exon 19 deletion) cells

**Fig. 5:**
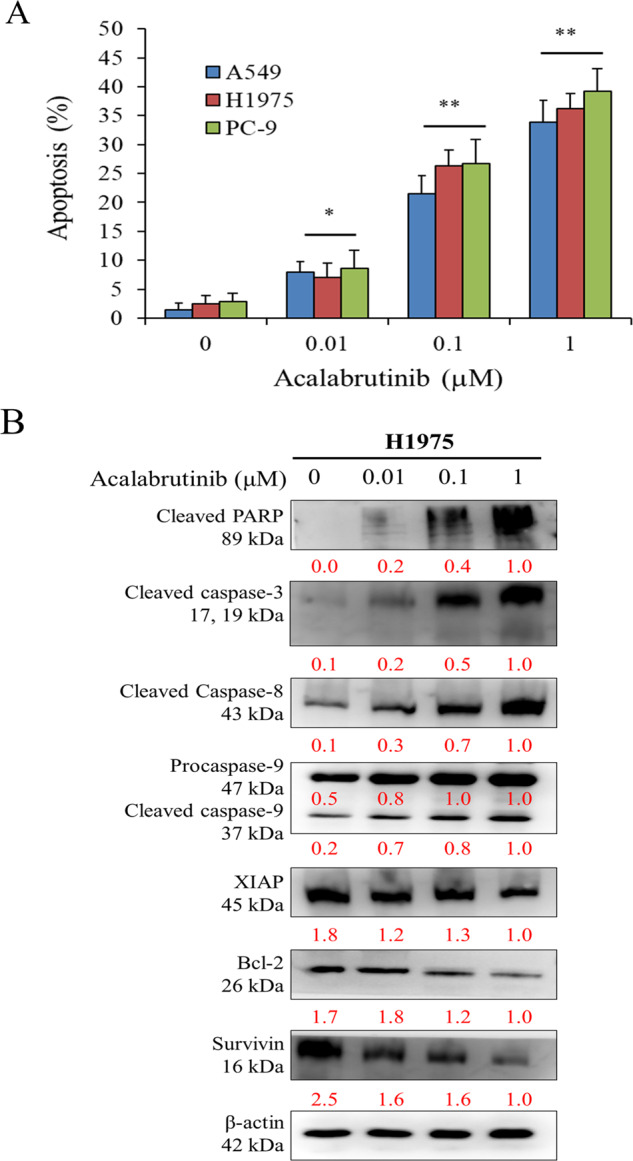
BTK inhibitor Acalabrutinib induces apoptosis in A549 (EGFR wild type), H1975 (EGFR T790M), and PC-9 (mutant EGFR with 19 Del) cells. **A** Effects of Acalabrutinib on apoptosis induction after treatment of Acalabrutinib at the indicated concentrations for 48 h. Apoptosis was determined by the annexin-V/PI assay. Bars represent mean ± SD. *p < 0.05, **p < 0.01. **B** Modulation of Acalabrutinib on apoptotic proteins in Gefitinib-resistant H1975 cells.

We further assessed the treatment effects of the BTK inhibitor Acalabrutinib on NSCLC cell lines with different EGFR status, namely A549 (wild-type EGFR), H1975 (EGFR T790M), and PC-9 (mutant EGFR with exon 19 deletion) cells. The results demonstrated that acalabrutinib induced apparent apoptosis in both Gefitinib-sensitive PC-9 and Gefitinib-resistant H1975 cells in a dose-dependent manner (Fig. 5A). We then examined the apoptosis-related proteins in the H1975 cells to verify that Acalabrutinib treatment caused a dose-dependent cleavage of Poly (ADP-ribose) polymerase, which is a hallmark of apoptosis. The activation of caspase-3, caspase-8, and caspase-9 as well as decreased expression of apoptosis inhibitors such as XIAP and Bcl-2 in a dose-dependent manner was also observed (Fig. 5B).

### Combined BTK/EGFR inhibition effectively suppresses tumor growth of Gefitinib-resistant Cells

**Fig. 6:**
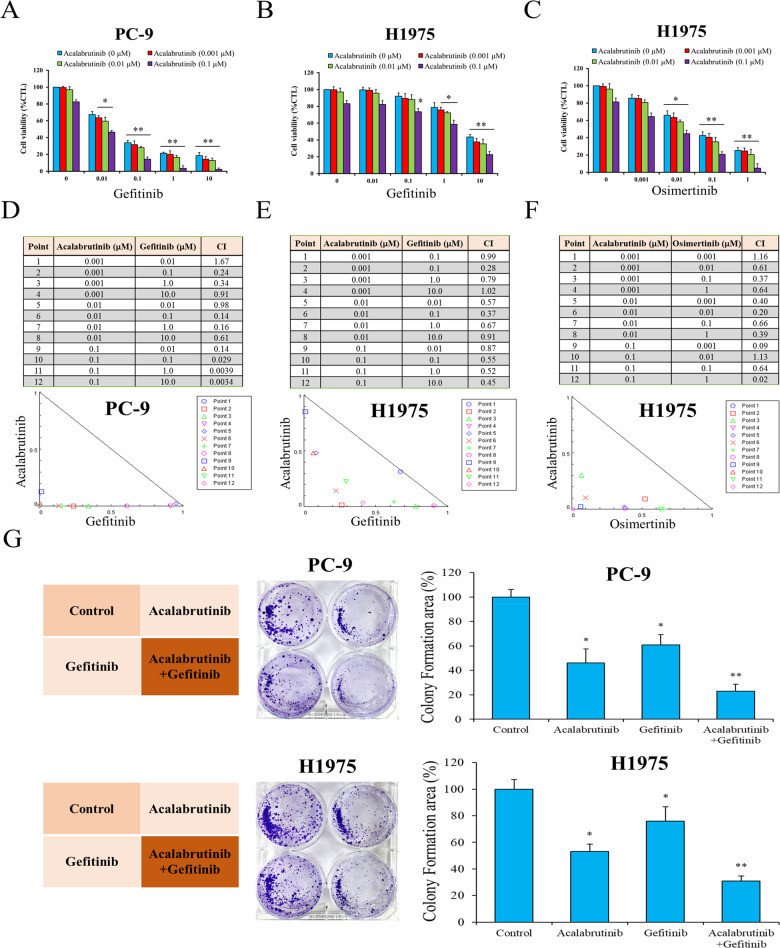
BTK inhibitor Acalabrutinib promotes the anti-proliferative effects of Gefitinib/Osimertinib in Gefitinib-sensitive PC-9 cells and Gefitinib-resistant H1975 cells. Cell viability after treatment with different concentrations of TKIs and Acalabrutinib was evaluated by SRB assay in PC-9 cells and H1975 cells. **A** PC-9: Gefitinib + Acalabrutinib; **B** H1975: Gefitinib + Acalabrutinib; **C** H1975: Osimertinib + Acalabrutinib. Combination index (CI) for drugs and isobolograms were used to illustrate the synergistic effects of each two drugs in PC-9 cells and H1975 cells. **D** PC-9: Gefitinib + Acalabrutinib; **E** H1975: Gefitinib + Acalabrutinib; **F** H1975: Osimertinib + Acalabrutinib. **G** Colony formation assay showed colony number of PC-9 and H1975 cells after treatment of Acalabrutinib with or without Gefitinib significantly decreased. *p < 0.05, **p < 0.01.

In addition to inducing apoptosis, BTK inhibitor Acalabrutinib also promoted the anti-proliferative effects of Gefitinib in both Gefitinib-sensitive PC-9 and Gefitinib-resistant H1975 cells (Fig. 6A, B). Notably, the cell viability of H1975 cells after Gefitinib treatment remained high but dropped dramatically after adding Acalabrutinib. Acalabrutinib also promoted the anti-proliferative effect of Osimertinib in H1975 cells (Fig. 6C). We then calculated CI for drugs and isobolograms were used to illustrate the synergistic effects of two drugs (Fig. 6D–F). To explore the effects of long-term exposure to single or combined drugs on anchorage-independent growth, we performed a soft-agar colony assay on PC-9 and H1975 cells. Two weeks after seeding, colony formation was remarkably suppressed by the combined treatment with Acalabrutinib and Gefitinib in both cells, and the degree of suppression in combination group was stronger than either Acalabrutinib or Gefitinib group (Fig. 6G). These results suggest that a combined treatment with BTK and EGFR TKI inhibitors may have some synergistic effect to suppress tumor growth, yet the mechanisms are still to be investigated.

### Acalabrutinib combination treatment inhibits NSCLC tumorigenesis, enhances sensitivity to Osimertinib, and improves survivability, in vivo

**Fig. 7:**
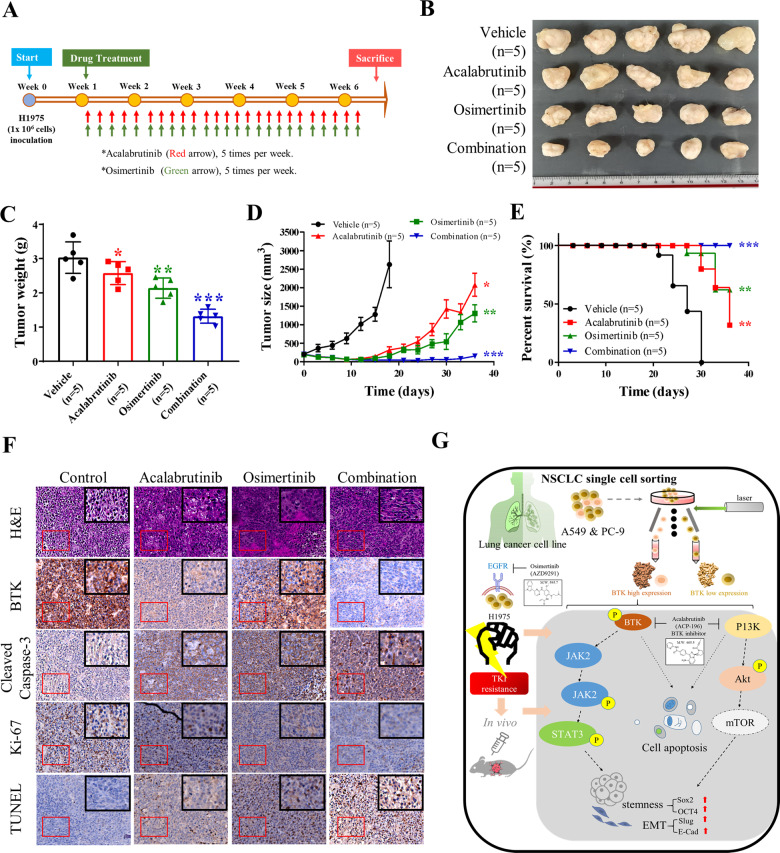
Acalabrutinib suppresses the EGFR TKI-resistant H1975 cells in vivo. **A** Experimental chart of in vivo studies. Photographs of NSCLC cells, H1975 (1 × 10^6^ cells/injection, subcutaneous) were injected into mice for establishing tumor xenograft model. When tumor size became palpable, mice were separated into four groups: Vehicle control, Osimertinib (20 mg/Kg, i.p injection, 5 times a week), Acalabrutinib (25 mg/kg, i.p. injection, 5 times a week), and combination of Osimertinib + Acalabrutinib. **B–D** Gross view, tumor weight and tumor size of H1975 tumors in the xenograft model. **E** Kaplan–Meier survival curve of the xenograft model. **F** Representative immunohistochemical staining images of differential expression of BTK, cleaved Caspase-3, Ki-67, and TUNEL in tumors extracted from the Osimertinib- and/or Acalabrutinib-treated NOD/SCID mice. *p < 0.05; **p < 0.01; ***p < 0.001. **G** Schematic illustration of current study. The in vitro studies demonstrated that Acalabrutinib suppressed SOX2 and STAT3/JAK2/Akt axis, leading to decreased stemness and EMT in A549 and PC-9 cells. The in vivo studies suggested that combination of Acalabrutinib and Osimertinib could effectively decrease tumorigenesis of H1975 cells.

To examine the translatability of our in vitro finding, we conducted xenograft model using EGFR TKI-resistant H1975 cells. Experimental chart of in vivo studies was shown in Fig. 7A. The tumor-bearing mice were treated with either 20 mg/kg Osimertinib or 25 mg/kg Acalabrutinib by intraperitoneal injection daily for 5 consecutive days per week until the end of week 6. However, the mice receiving 20 mg/kg Osimertinib plus 25 mg/kg Acalabrutinib had injection only three times a week. The inhibitory effect of tumor growth was most significant in the combination group, followed by Osimertinib only, Acalabrutinib only, and the vehicle group (Fig. 7B–D). The mice group receiving the combination treatment showed the highest survival ratios followed by Osimertinib only and Acalabrutinib only, whereas vehicle mice showed the lowest survival ratios (Fig. 7E). Furthermore, the immunohistochemical analyses (Fig. 7F) indicated the lowest BTK staining in the tumor section from the combination group, followed by Acalabrutinib only group, while BTK staining remained high in both Osimertinib only group and vehicle group. The cellular apoptosis markers (cleaved caspase-3 and TUNEL) and proliferation marker (Ki-67) were shown as well, and they were compatible with the tumor changes in xenograft model. Finally, a schematic illustration was shown in Fig. 7G. The in vitro studies demonstrated that Acalabrutinib suppressed SOX2 and STAT3/JAK2/Akt axis, leading to decreased stemness and EMT in A549 and PC-9 cells. The in vivo studies suggested that combination of Acalabrutinib and Osimertinib could effectively decrease tumorigenesis of H1975 cells.

## Discussion

Most patients with NSCLC that receive EGFR TKIs experience disease progression as a result of acquired resistance within approximately one year. The mechanisms of acquired resistance to EGFR TKIs have been gradually elucidated, and target gene modification (most commonly T790M), alternative pathway activation, and histological or phenotypic transformation have been reported [22–26]. To manage progressive disease after the use of EGFR TKIs, certain treatments were proposed, such as Osimertinib for EGFR T790M-positive tumors and platinum-based chemotherapy or the application of other targetable agents for *EGFR* T790M-negative tumors [27]. However, most treatments were unsatisfactory, and Osimertinib resistance also became a recognized problem [28, 29]. Therefore, investigations into new treatment strategies beyond the abovementioned approaches are urgently required.

Our study demonstrated that BTK plays a vital clinicopathologic role. High BTK expression was associated with poor tumor differentiation, advanced pathologic stage, lymph node metastasis, and large maximum tumor size. The patients with NSCLC with a high BTK expression also had poor OS and disease-free survival (Fig. 1). These results were generally compatible with the results of studies on solid tumors [15,16,] and one study on NSCLC [30]. However, a contradictory report stated that high BTK expression correlated negatively with clinical stages and distant metastasis, but was instead associated with a favorable prognosis [31]. This discrepancy may be a result of different methodologies. The report was derived from the Cancer Genome Atlas database using genomic profiles, in contrast to our study, which was derived from a patient cohort using immunohistochemistry. The genomic profiles of NSCLC tumors likely contained information from mixed cells rather than pure tumor cells; therefore, the real BTK expression in NSCLC cells could not be accurately determined. Additionally, the report did not sufficiently explain why high BTK expression resulted in a more favorable prognosis.

In addition to the immunohistochemical evaluation, our in vitro studies revealed that a high BTK expression in NSCLC cells promoted migration and invasion, upregulated the expression of stemness genes OCT4 and SOX2, and enhanced EMT by increasing slug and vimentin and decreasing E-cadherin (Fig. 2). Regarding the regulating mechanisms associated with BTK expression, we determined that the JAK2/STAT3 pathway may play a vital role. The p-STAT3 was upregulated in BTK^pos^ cells, and treatment with the BTK inhibitor Acalabrutinib suppressed the expression of p-JAK2, p-STAT3, and p-Akt in these cells in a dose-dependent manner. The phenotypic presentations of migration, colony formation, and sphere formation were also consistently suppressed by the Acalabrutinib treatment (Fig. 3C–E). These data were compatible with other reports that have focused on the relationship between BTK and the JAK/STAT3 pathway in both malignant [32–34] and benign [35, 36] conditions. However, to our knowledge, this study is the first to document such a relationship in NSCLC.

We evaluated the roles of BTK in two different EGFR-TKI-resistant NSCLC cells. The first was the PC-9/GR cells, where the PC-9 cells acquired Gefitinib resistance after selection. A knockdown of BTK in the PC-9/GR cells induced significantly more cell death after Gefitinib treatment (Fig. 4A, C). Conversely, overexpression of BTK in the PC-9 cells, which were EGFR-TKI-sensitive cells, resulted in less cell death after Gefitinib treatment (Fig. 4B, D). The expression of CSC markers (SOX2, KLF4, NANOG, and CD133) was significantly higher in PC-9/GR and PC-9/pBTK-N1 cells (Fig. 4E, F), indicating that BTK may regulate the stemness of NSCLC cells to acquire the phenotype of Gefitinib resistance. The relationship between CSCs and drug resistance has already been thoroughly investigated [37, 38], and our data supported the potentially vital role of BTK in the drug resistance of NSCLC cells, regardless of their EGFR mutation status.

The second EGFR-TKI-resistant NSCLC cells were H1975, which harbored the T790M mutation. We demonstrated that Acalabrutinib treatment caused considerable apoptosis of H1975 cells, comparable to that of A549 cells (EGFR wild type) and PC-9 cells (EGFR exon 19 deletion). Further investigation of apoptosis-related proteins in H1975 cells validated the effect of Acalabrutinib (Fig. 5B). Subsequently, we evaluated the inhibitory effect of Acalabrutinib on the cell proliferation and colony formation of PC-9 and H1975 cells. Acalabrutinib alone exhibited an inhibitory effect on both cell proliferation and colony formation, and Acalabrutinib in combination with Gefitinib resulted in increased suppression (Fig. 6). Although the detailed mechanisms remain unclear, this study evidenced the synergistic effect of combined Acalabrutinib and Gefitinib treatment in NSCLC. Combination treatment is a useful strategy in treating cancers, including lung cancer [39–42], and our data suggested that Acalabrutinib serves as a novel component in future combination therapy for NSCLC.

In this study, we used Acalabrutinib to achieve a more effective inhibition of BTK. Also known as ACP-196, Acalabrutinib is a second-generation BTK inhibitor that was designed to be more potent and selective than ibrutinib [43]. In 2019, Acalabrutinib was approved by the US Food and Drug Administration for the treatment of adult patients with chronic lymphocytic leukemia and small lymphocytic leukemia. According to reports, it failed to inhibit the activities of platelets and many kinases, including EGFR, ITK, and TEC [44–46]. Once the treatment effects of Acalabrutinib in NSCLC are further validated, this heightened safety profile will widen its applicability. Furthermore, we evaluated the therapeutic role of Acalabrutinib using an EGFR TKI-resistant xenograft model. H1975 (*EGFR* T790M) cells are well known to respond to the third-generation EGFR TKI, Osimertinib. We then compared the treatment effect of Acalabrutinib with Osimertinib and investigated their combination effect. We found that combination of Acalabrutinib and Osimertinib provided the most pronounced reduction in mice tumor burden, followed by Osimertinib alone. Notably, the combination of Acalabrutinib and Osimertinib exhibited significant tumor growth inhibition in vivo, indicating that Acalabrutinib could potentiate mice-bearing EGFR TKI-resistant tumors to Osimertinib treatment (Fig. 7). This also worked in concert with our in vitro data which were shown in Fig. 6.

In conclusion, our current study documented that BTK regulates stemness, EMT and Gefitinib resistance in NSCLC cells, thus results in poor prognosis of NSCLC patients. Our findings also suggested that treatment of Acalabrutinib with or without Gefitinib can effectively suppress the resistant phenotypes of NSCLC cells. Acalabrutinib suppressed SOX2 and STAT3/JAK2/Akt axis activation leading to decreased stemness and EMT. Moreover, we showed the synergic effect of combining Acalabrutinib and Osimertinib in the treatment of H1975 tumor in vivo. Therefore, inhibition of BTK, favorably combining with other therapeutics, could be a promising strategy in the treatment of NSCLC.

## Supplementary information

Supplementary Information
