# Supplementary material for: Bruton’s tyrosine kinase (BTK) mediates resistance to EGFR inhibition in non-small-cell lung carcinoma
Source: Oncogenesis. 2021 Jul 27;10(7):56. doi: 10.1038/s41389-021-00345-8 (PMC8316404; doi:10.1038/s41389-021-00345-8)
Supplement: Supplementary file 1 — Supplementary Information [file 41389_2021_345_MOESM1_ESM.docx]

**SUPPLEMENTARY INFORMATION**

**Bruton’s tyrosine kinase (BTK) mediates resistance to *EGFR* inhibition in non-small cell lung carcinoma**

Chi-Tai Yeh^1,2,3^, Tzu-Tao Chen^4,5^, Pamungkas Bagus Satriyo^1,6,7^, Chun-Hua Wang^8,9^, Alexander T.H. Wu^10^, Tsu-Yi Chao^2,11^, Kang-Yun Lee^4,5,11^, Michael Hsiao^12^, Liang-Shun Wang^13^, Kuang-Tai Kuo^13,14^

^1^ Department of Medical Research and Education, Shuang Ho Hospital, Taipei Medical University, New Taipei City 23561, Taiwan

^2^ Division of Hematology & Oncology, Department of Medicine, Shuang Ho Hospital, Taipei Medical University, New Taipei City 23561, Taiwan

^3^ Department of Medical Laboratory Science and Biotechnology, Yuanpei University of Medical Technology, Hsinchu City 30015, Taiwan

^4^ Division of Pulmonary Medicine, Department of Internal Medicine, Shuang Ho Hospital, Taipei Medical University, New Taipei City 23561, Taiwan

^5^ Division of Pulmonary Medicine, Department of Internal Medicine, School of Medicine, College of Medicine, Taipei Medical University, Taipei 110, Taiwan

^6^ Faculty of Medicine Public Health and Nursing, Universitas Gadjah Mada, Yogyakarta 55281, Indonesia

^7^ Department of Pharmacology and Therapy, Faculty of Medicine Public Health and Nursing, Universitas Gadjah Mada, Yogyakarta 55281, Indonesia.

^8^ Department of Dermatology, Taipei Tzu Chi Hospital, Buddhist Tzu Chi Medical Foundation, New Taipei City 23561, Taiwan

^9^ School of Medicine, Buddhist Tzu Chi University, Hualien, Taiwan

^10^ The Ph.D. Program for Translational Medicine, College of Medical Science and Technology, Taipei Medical University, Taipei 110, Taiwan

^11^ Graduate Institute of Clinical Medicine, College of Medicine, Taipei Medical University, Taipei 110, Taiwan

^12^ Genomics Research Center, Academia Sinica, Taipei, Taiwan

^l3^ Division of Thoracic Surgery, Department of Surgery, Shuang Ho Hospital, Taipei Medical University, New Taipei City 23561, Taiwan

^14^ Division of Thoracic Surgery, Department of Surgery, School of Medicine, College of Medicine, Taipei Medical University, Taipei 110, Taiwan

These authors contributed equally: Chi-Tai Yeh and Tzu-Tao Chen

*Corresponding author:

Dr. Kuang-Tai Kuo

Division of Thoracic Surgery, Department of Surgery, Shuang Ho Hospital, Taipei Medical University, New Taipei City 23561, Taiwan; Tel: +886-2-2490088 ext. 8119; Fax: +886-2-2248-0900. E-mail addresses: [ktkuo@tmu.edu.tw](mailto:ktkuo@tmu.edu.tw)

*Running title:* *BTK promotes resistance to* EGFR*-*TKI *in NSCLC*

 **Supplementary Table S1.** The commercial antibodies list

**Supplementary Table S2.** The sequences of the RT-PCR primers list.

| **Gene** | **Forward Sequence (5' → 3')** | **Reverse Sequence (5' → 3')** |
| --- | --- | --- |
| BTK | GGTGGAGAGCACGAGATAAA | CCGAGTCATGTGTTTGGAATAC |
| E-cadherin | TTTGTACAGATGGGGTCTTGC | CAAGCCCACTTTTCATAGTTCC |
| KLF4 | CCTTTCAGTGCCAGAAGT | ACTACGTGGGATTTAAAAGTGC |
| SOX2 | AGGGCTGGACTGCGAACTG | TTTGCACCCCTCCCAATTC |
| OCT4 | CTTGCTGCAGAAGTGGGTGGAGGAA | CTGCAGTGTGGGTTTCGGGCA |
| NANOG | ATGGAGGAGGGAAGAGGAGA | GATTTGTGGGCCTGAAGAAA |
| Slug | CCTGGTCAAGAAGCATTTCAA | GCCCCAAAGATGAGGAGTATC |
| Vimentin | TGGCACGTCTTGACCTTGAA | GGTCATCGTGATGCTGAGAA |

**Supplementary Table S3.** Univariate and multivariate analysis of BTK expression in the NSCLC cohort.

|  | **Univariate** | | | | **Multivariate** | | | | |
| --- | --- | --- | --- | --- | --- | --- | --- | --- | --- |
|  | **HR** | **95%** | | **P-value** | **HR** | **95%** | | **P-value** |  |
| **Age** | 1.591 | 0.899 | 2.816 | 0.1108 | 3.135 | 8.850 | 1.116 | 0.030 |  |
| **Gender** | 0.594 | 0.337 | 1.047 | 0.0719 | 0.424 | 1.319 | 0.136 | 0.138 |  |
| **TNM Stage** | 4.151 | 2.178 | 7.912 | <0.001 | 2.681 | 12.821 | 0.562 | 0.216 |  |
| **Subtype** | 0.235 | 0.118 | 0.468 | <0.001 | 0.233 | 1.449 | 0.038 | 0.118 |  |
| **BTK expression** | 3.626 | 1.533 | 8.57 | 0.0029 | 5.155 | 23.256 | 1.152 | 0.032 |  |


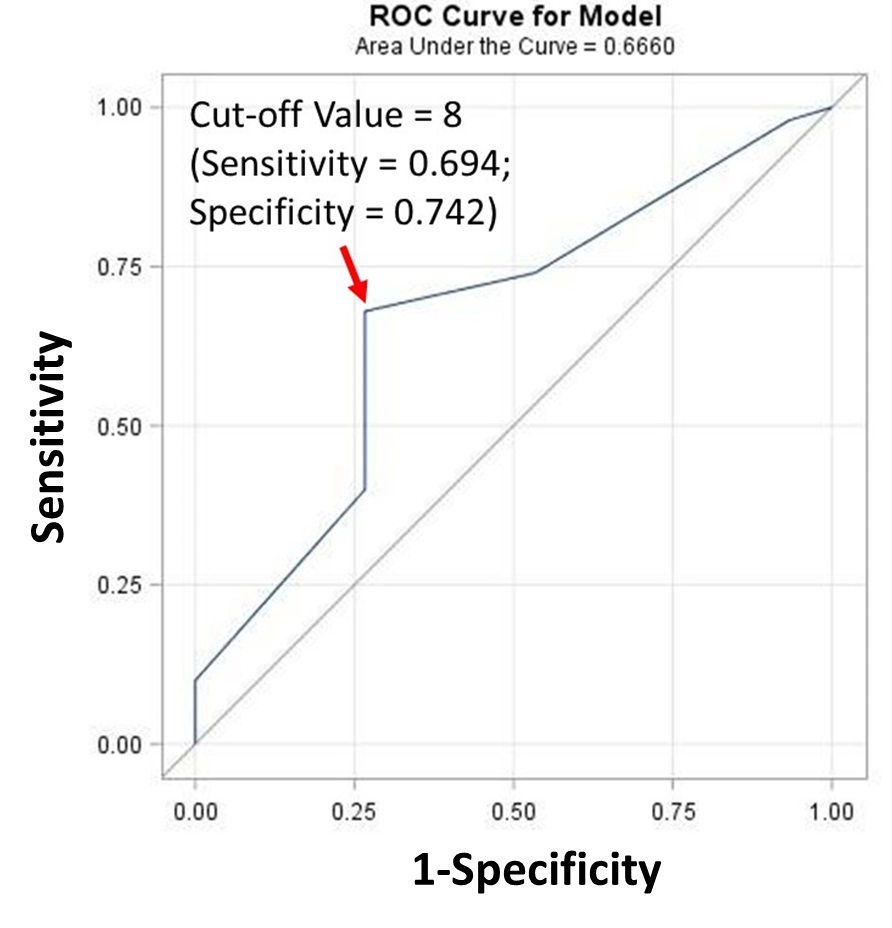


**Supplementary Figure S1.** ROC curve analysis shows the optimal cut-off point is 8.0 with 0.694 sensitivity and 0.742 specificity.


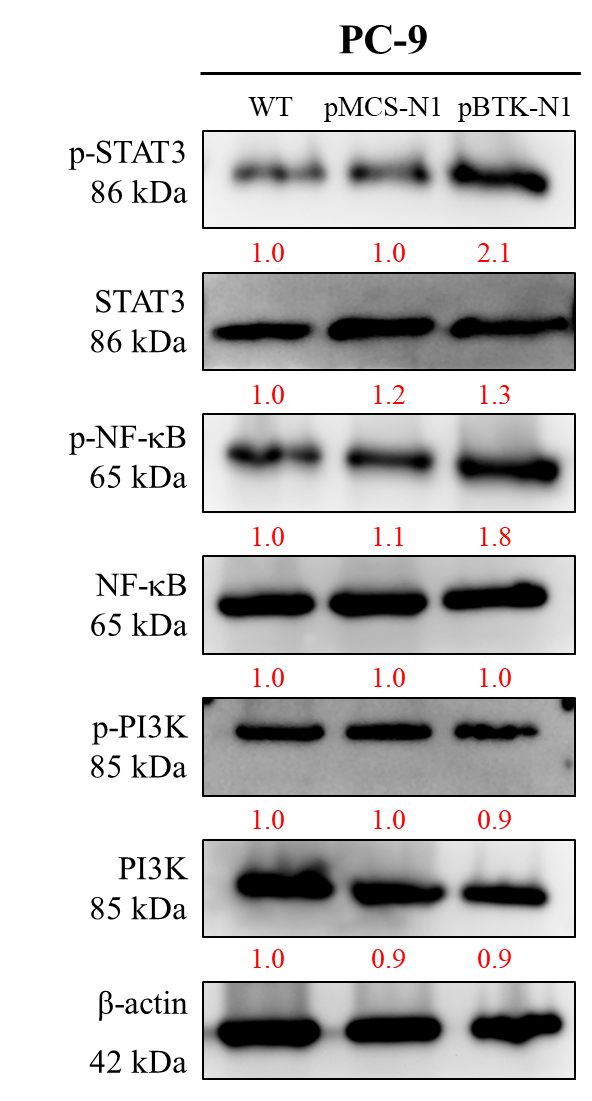


**Supplementary Figure S2.** The downstream activated pathway in BTK overexpression.
